# Alternative diagnoses of suspected paraneoplastic neurological syndromes: a population-based study

**DOI:** 10.1007/s00415-026-13737-w

**Published:** 2026-03-11

**Authors:** Alberto Vogrig, Francesco Bax, Gian Luigi Gigli, Andrea Bernardini, Alessandro Marini, Michele Rana, Paolo Passadore, Daniele Bagatto, Francesco Curcio, Sergio Muñiz-Castrillo, Jérôme Honnorat, Martina Fabris, Mariarosaria Valente

**Affiliations:** 1 Clinical Neurology, Department of Head-Neck and Neuroscience, Azienda Sanitaria Universitaria Friuli Centrale (ASUFC), Udine, Italy; 2 Department of Medicine (DMED), University of Udine, Udine, Italy; 3 Neurology Unit, Hospital of Gorizia, Azienda Sanitaria Universitaria Giuliano Isontina (ASUGI), Gorizia, Italy; 4 Neurology Unit, Ospedale Santa Maria Misericordia, Rovigo, Italy; 5 Department of Diagnostic Imaging, Unit of Neuroradiology, Azienda Sanitaria Universitaria Friuli Centrale (ASUFC), Udine, Italy; 6 Institute of Clinical Pathology, Department of Laboratory Medicine, Azienda Sanitaria Universitaria Friuli Centrale (ASUFC), Udine, Italy; 7 Neurology Department, Hospital Universitario 12 de Octubre, Instituto de Investigación Sanitaria Hospital 12 de Octubre (Imas12), Madrid, Spain; 8 Reference Centre for Paraneoplastic Neurological Syndromes and Autoimmune Encephalitis, Hospices Civils de Lyon, Neurological Hospital, Bron, France; 9 MeLiS-UCBL-CNRS UMR 5284-INSERM U1314, Université Claude Bernard Lyon 1, Lyon, France

**Keywords:** Paraneoplastic neurologic syndromes, Autoimmune encephalitis, Limbic encephalitis, Misdiagnosis, Mimics

## Abstract

**Objective:**

The diagnosis of paraneoplastic neurologic syndromes (PNS) requires exclusion of alternative etiologies. However, the spectrum of alternative diagnoses encountered during PNS workup (“better explanations”) has not been systematically examined. We aimed to characterize this spectrum.

**Methods:**

Retrospective population-based study in Friuli-Venezia-Giulia (Italy). Among 878 consecutive patients tested for PNS antibodies at a centralized tertiary center serving three hospitals (2009–2017), alternative diagnoses were identified. Results of a logistic regression model were used to develop the *PNS DDx Score*.

**Results:**

Among 661 patients with alternative diagnoses, median age was 66 years, and 51% were male. CNS involvement predominated in 397 patients (60%), peripheral in 215 (33%), and extra-nervous system in 49 (7%). Etiologic diagnosis was reached in 269 with central involvement, including degenerative (97, 36%), autoimmune (47, 17%), and vascular (37, 14%). In peripheral presentations, a diagnosis was reached in 112 and included autoimmune (51, 45%), toxic-metabolic (40, 36%), and vasculitis (11, 10%). PNS antibody testing increased by 96% from 2009 to 2017; diagnostic yield remained low (7% vs. 8%). Intermediate-risk or other syndromes (OR 7.97, CI95% 3.07–23.80; OR 192.82 CI95%, 66.11–739.59) and low-risk/absence of tumor (OR 6.90, CI95% 2.75–18.92) were associated with alternative diagnoses, whereas increasing age (OR 0.95, CI95% 0.92–0.97) was inversely associated. These variables (age, syndrome, and tumor type) were incorporated in the *PNS DDx Score*.

**Conclusion:**

Alternative diagnoses during PNS workup are common in patients < 55 years and those without high-risk syndromes. The *PNS DDx Score* can be used to identify patients at risk of misdiagnosis.

**Supplementary Information:**

The online version contains supplementary material available at 10.1007/s00415-026-13737-w.

## Introduction

Paraneoplastic neurological syndromes (PNS) are rare neuroimmune disorders triggered by a systemic malignancy [1, 2], with incidence rates ranging from 1.6 per million person-years in France [3], 2.6 in the Netherlands [4], and 6 in Olmsted County (Minnesota, USA) [5], to 8.9 in northeastern Italy [6].

Given their rarity, a broad spectrum of neurological alternative diagnosis must be considered when approaching patients with suspected PNS. Exclusion of other common causes of the neurological dysfunction (e.g., direct invasion of cancer, side-effects of chemotherapy, infections, and metabolic or nutritional deficits) must be pursued before making a diagnosis of PNS [7]. The concept of excluding alternative causes is a key element of both PNS and autoimmune encephalitis (AE) diagnostic criteria [2, 8, 9]; however, little has been done to gather information on diseases that are encountered during PNS workup in clinical practice. In the diagnostic evaluation, search for neuronal antibodies (Abs) can be very helpful, but also harbors several challenges: a subgroup of patients with classic (“high-risk”) PNS phenotypes can be Ab-negative [2, 6, 10], while in other reportedly seropositive cases, the presence of atypical clinical features may underlie a false-positive result if comprehensively investigated [11–14]. Therefore, an accurate diagnostic process is needed to identify other potentially treatable diseases, and to avoid the unnecessary regular cancer screening as well as the potentially harmful initiation of immunosuppressants drugs [13, 14].

Herein, we conducted a post hoc analysis on a population-based study aimed at evaluating the spectrum of alternative diagnoses (“better explanations”) encountered during PNS workup in the Friuli-Venezia Giulia Region (Italy). In addition, we provide recommendations to avoid the risk of misdiagnosis based on the most common alternative etiologies encountered.

## Methods

### Study population

The present study stemmed from a previous 9-year (2009–2017) retrospective, observational, population-based study conducted by the authors which defined the epidemiology of PNS in the Italian provinces of Udine, Pordenone, and Gorizia in the Friuli-Venezia Giulia Region, Italy (983,190 people as of January 1, 2017) [6]. This study analyzed 878 consecutive patients screened for the presence of neuronal Abs at the Department of Laboratory Medicine, Udine University Hospital using the methodology previously described [6]. The clinical characteristics, antibody associations, as well as oncological features of patients with a final diagnosis of definite PNS (*n* = 89; median age, 68 years; 52% women) were described in the original publication [6]. Additionally, a second group received a diagnosis of possible PNS (*n* = 128; median age, 69 years; 42% women). Finally, a third group (*n* = 661) received a final alternative diagnosis not attributable to PNS and constitutes the focus of the present study.

### Inclusion criteria

Inclusion criteria of the present study were: (1) a prior clinical suspicion of PNS prompting neuronal Ab testing; (2) negative laboratory testing for neuronal Abs fulfilling PNS criteria at Udine University Hospital using 2 complementary techniques (tissue-based and antigen-specific assays), or, in the case of indeterminate results, confirmation of a final negative result at the French Reference Center for PNS and AE; and (3) establishment of a final diagnosis providing a better explanation for the patient’s neurologic symptoms and not attributable to a paraneoplastic etiology. Such alternative diagnoses were based on objective diagnostic testing (e.g., histopathologic confirmation of a brain tumor), established clinical diagnostic criteria with or without supportive ancillary investigations (e.g., functional neurologic disorder), or, in patients without a specific alternative neurologic diagnosis, prolonged systematic cancer screening remaining negative for at least 5 years.

After comprehensive clinical evaluation by neurologists with expertise in autoimmune neurology (AV, AB, and AM), patients with an alternative diagnosis of a non-PNS condition (*n* = 661, 75%) were identified. Relevant data (age, sex, clinical, and paraclinical features), stored in an informatics database, were examined in detail by AV and FB, assessing the type and frequency of alternative diagnoses, the clinical features, and the results of paraclinical studies. In addition, magnetic resonance imaging (MRI) studies were reviewed by an expert neuroradiologist (DB).

### Alternative diagnoses classification

Data on alternative diagnoses were categorized according to (1) clinical presentation, including the predominant location of the neurological dysfunction classified as either central nervous system (CNS), peripheral nervous system (PerNS) disorders, or extra-nervous system (Extra-NS) involvement when no objective evidence of involvement of the nervous system was documented, and (2) by etiologic diagnosis, whenever found. Etiological categories of the alternative neurological diagnoses were defined a priori as follows: degenerative, autoimmune, vascular, neoplastic, toxic-metabolic, structural, infectious, psychiatric, genetic, and traumatic. Autoimmune non-PNS disorders included also patients with AE (diagnosed following the 2016 criteria [9]) without identified tumor after a 5-year follow-up. Conversely, patients with vasculitis were included in the category of vascular disorder. When a tumor was found during oncological screening, but the patient’s symptoms were judged to be unrelated to cancer (e.g., Parkinson’s disease in a patient with prostate cancer, or dementia in a patient with skin cancer), it was categorized as “low-risk tumor”. This classification applied when the tumor did not belong to the typical oncological associations described in the updated PNS diagnostic criteria [2], including lung cancer (both small-cell and non-small-cell), thymoma, breast and ovarian cancers, testicular cancer, and Hodgkin lymphoma.

### Diagnostic yield of PNS antibody testing

Yearly and cumulative (9-year) determination of the ratio between number of patients with a diagnosis of definite PNS and the total number of patients tested for “high-risk” and “intermediate-risk” neuronal Abs (diagnostic yield) were obtained. Corresponding 95% confidence intervals (95% CIs) were calculated using the Clopper–Pearson method for binomial distributions.

### Statistical analysis

Variables were summarized with descriptive statistics. Absolute frequencies and percentages were used for categorical variables, median, and range for continuous variables. To assess temporal variations in diagnostic yield over time (2009–2017), Cochrane–Armitage test for linear trends was applied (p value < 0.05 was considered as statistically significant).

### *PNS DDx Score* development

A clinical score (*PNS DDx Score*) was built to predict the probability of an alternative diagnosis other than PNS to help with the clinical decision of undergoing second-level diagnostics when a PNS is suspected. Only definite PNS diagnoses on one side and alternative diagnoses on the other (661 patients with an alternative diagnosis and 89 with a definite PNS) were used in the derivation phase to ensure reliance on definite diagnostic categories.

First, the study population was randomly split into a derivation and a validation cohort (0.7/0.3 ratio). A logistic regression model assessing the odds ratio (OR) and 95% confidence interval (CI) of an alternative vs. definite PNS diagnosis was then built including clinically relevant variables selected a priori: age, sex, presenting neurological syndrome (high-risk, intermediate-risk, or other) [2], and tumor status (i.e., no tumor/low-risk tumor and high-risk tumor). Complete and quasicomplete separation among categorical variables was assessed using the *detectseparation* package to evaluate whether any independent variable in the score model perfectly discriminated between alternative vs. definite PNS diagnosis. Calibration curves were then plotted and Brier score calculated. Statistically significant variables were retained, and beta coefficients were used to build the score [15]. Subsequently, score accuracy in discriminating alternative vs. definite PNS diagnosis was validated using the area under the receiver-operating characteristic curve (AUC of ROC) both in the discovery and validation cohort, and to avoid data overfitting, we performed a 2000 internal bootstrap resampling. Sensitivity, specificity, negative predictive value, positive predictive value, and accuracy were also calculated. Statistical analysis was conducted with R (version 4.4.0) using the *pROC* package.

### Standard protocol approvals, registrations, and patient consents

This study was performed in line with the principles of the Declaration of Helsinki and was approved by the Institutional Review Board of the University of Udine (IRB DAME protocol number: 36/IRB_18). Being this a non-interventional, retrospective study, patient consent was not required. Anonymized data were collected in a secure (encrypted and password-protected) database. Patients were treated according to best clinical practice, independently of inclusion in the study. In addition, data collection and study results had no impact on patient care.

### Data availability statement

Anonymized data not published within this article will be made available by request from any qualified investigator.

## Results

Among 661 patients included with an alternative diagnosis, median age was 66 years (range: 2–91) and 335 patients (51%) were male. CNS was the site of predominant involvement in 397 (60%), PerNS in 215 (33%), and extra-NS in 49 (7%).

### Alternative diagnoses with central nervous system involvement

**Fig. 1:**
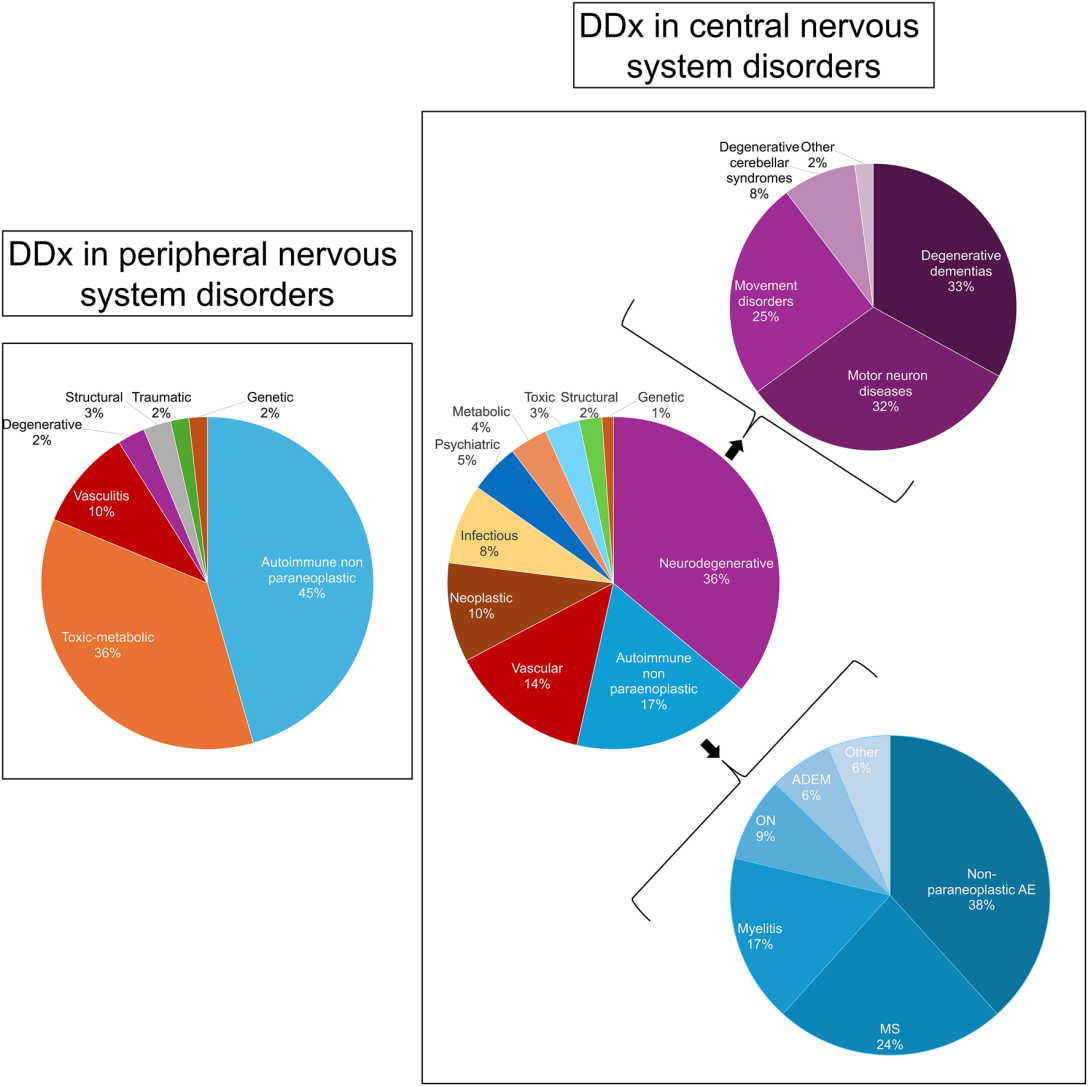
Alternative neurological diagnoses encountered (“better explanations”) during PNS workup. Note that the graphs refer only to cases where a final diagnosis was established (*n* = 269 for patients with central nervous system manifestations and *n* = 112 for those with a peripheral nervous system presentation)

During the study period, an alternative diagnosis was established in 269/397 patients with CNS involvement (68%), while it remained unknown in 128 (32%; e.g., epilepsy of unknown etiology). When a final diagnosis was reached (*n* = 269), the most frequent etiologies were, in order of frequency: degenerative 97 (36%), autoimmune 47 (17%), vascular 37 (14%), neoplastic 26 (10%), infectious 21 (8%), psychiatric 13 (5%), and metabolic 10 (4%), followed by other less frequent diagnoses in 18 (7%) (Fig. 1).I have uploaded a new version of Figure 1 identical to the previous one, with slightly larger labels to improve readability.

Among neurodegenerative conditions tested for PNS Abs (*n* = 97), the most common diseases were represented by degenerative dementias (*n* = 32, 33%), motor neuron diseases (MND) (*n* = 31, 32%), and degenerative movement disorders (*n* = 24, 28%). In the group of degenerative dementias (*n* = 32), detailed diagnoses were reached in 18 cases (56%), while in 14 (44%), the follow-up and cerebrospinal fluid (CSF) biomarkers permitted only a diagnosis of unspecified degenerative dementia. Dementia diagnoses included Alzheimer’s disease (AD) (*n* = 5), mixed dementia (vascular and AD) (*n* = 3), frontotemporal dementia (*n* = 3), Lewy body dementia (*n* = 3), Creutzfeldt–Jakob disease (CJD) (*n* = 3), and primary progressive aphasia (*n* = 1).

In the group of MND (*n* = 31), the majority of the cases (*n* = 22, 71%) were atypical for classic amyotrophic lateral sclerosis (ALS) and the clinical features that led to neuronal Ab testing were the presence of increased CSF protein content (*n* = 5), young age at onset (< 50 years) (*n* = 4), concomitant sensory disturbances (*n* = 4), infrequent clinical variants (flail arm syndrome *n* = 2, Hirayama disease *n* = 1, primary lateral sclerosis *n* = 1), presence of a concomitant systemic tumor (*n* = 3), or presence of associated cognitive disturbances at onset (*n* = 2).

Patients with degenerative movement disorders (*n* = 24) included cases with atypical parkinsonism (*n* = 11, including 4 patients with multiple system atrophy [MSA]), idiopathic Parkinson’s disease (PD) (*n* = 9), chorea (*n* = 3, including 2 cases of Huntington’s disease), and pure autonomic failure (PAF) (*n* = 1). The most frequent reason for neuronal Ab testing in patients with movement disorders was the detection of a systemic tumor (*n* = 6, 25%), followed by the presence of autonomic dysfunction (*n* = 5, 21%).

Patients with an autoimmune etiology (*n* = 47) comprised those with non-paraneoplastic AE (*n* = 18, 38%), multiple sclerosis (MS) (*n* = 11, 23%), myelitis (*n* = 8, 17%), optic neuritis (*n* = 4, 8%), acute disseminated encephalomyelitis (ADEM) (*n* = 3, 6%), and less common diseases (cerebellitis, Sydenham chorea, and autoimmune hemichorea in 1 each). A subgroup of AE cases harbored neuronal surface Abs, including 4 patients with LGI1-Abs and 2 with CASPR2-Abs, all 6 without an associated tumor. The presence of cancer was detected in a minority of the other patients with autoimmune diseases mimicking PNS (*n* = 4, 9%).

Vascular disorders (*n* = 37) included ischemic stroke (*n* = 17, 46%), vasculitis (*n* = 10, 27%), subcortical vascular encephalopathy (*n* = 5, 14%), spinal infarction (*n* = 3, 8%), and hemorrhagic stroke (*n* = 2, 5%). A tumor was detected in 6 of them (16%). Among the 10 cases of vasculitis, 6 were primary angiitis of the central nervous system (PACNS), while 4 were systemic vasculitis with involvement of the CNS (Churg–Strauss syndrome, Behçet disease, anti-neutrophil cytoplasmic antibodies (ANCA)-associated vasculitis, and leukocytoclastic vasculitis in 1 each).

**Fig. 2:**
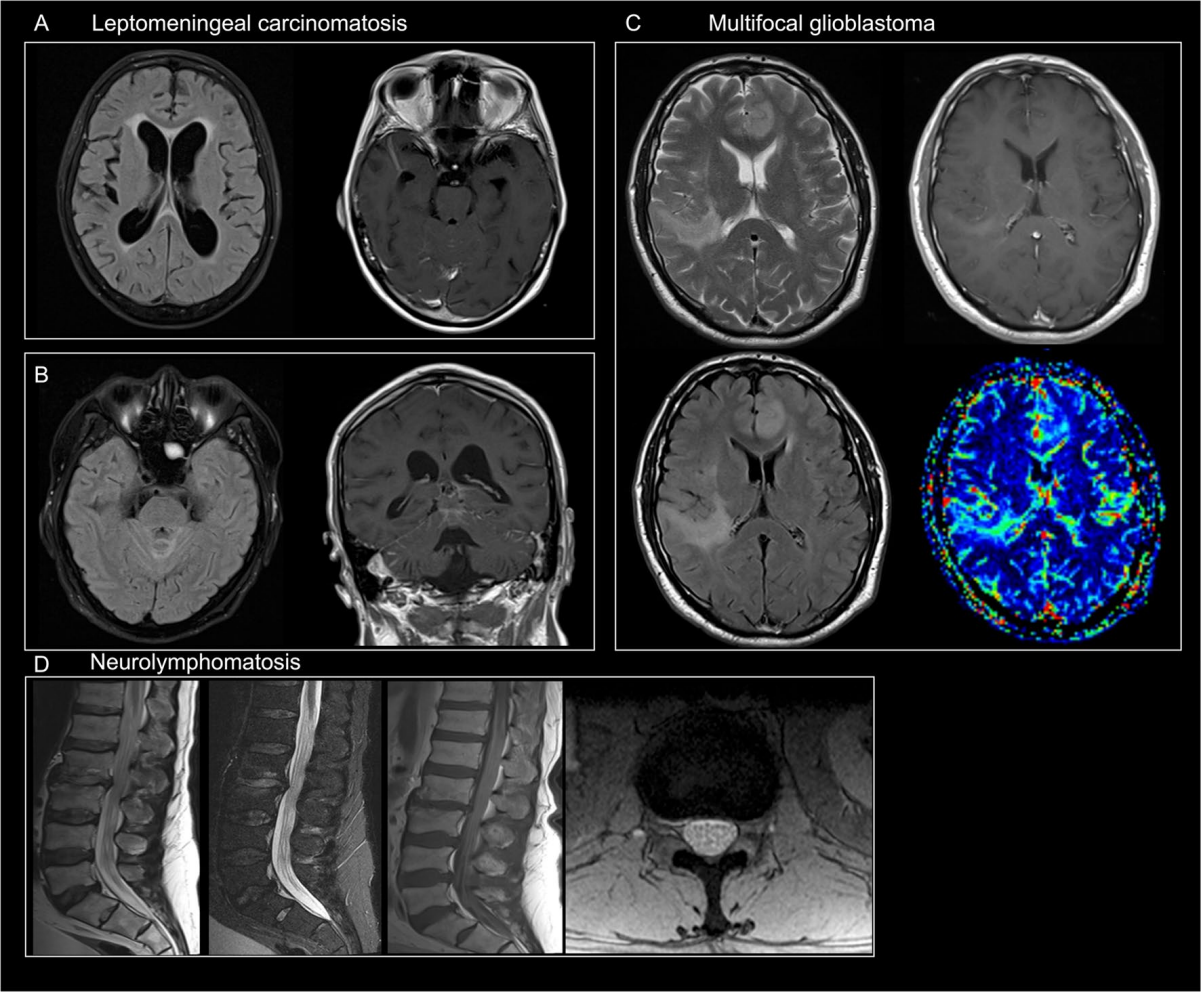
Imaging examples of patients who were initially thought to have PNS but later had an alternative neoplastic diagnosis. **A** and **B** Two distinct cases of leptomeningeal carcinomatosis are shown. In case A, axial FLAIR images demonstrated moderate dilation of the lateral ventricles, associated with periventricular hyperintensities, particularly at the level of the frontal horns. Post-gadolinium SE T1-weighted images revealed enhancement along the vermis and cerebellar sulci. In case B, a bright signal along the cerebellar sulci was seen on axial FLAIR images, while diffuse leptomeningeal enhancement was observed on coronal post-gadolinium SE T1-weighted images. **C** Multiple hyperintense lesions on T2-weighted and FLAIR images involved both the cortex and white matter of the left frontal lobe and right temporal lobe. No contrast enhancement was seen on post-gadolinium SE T1-weighted images; however, perfusion evaluation revealed a significant increase in relative cerebral blood volume (rCBV) in the same areas. The patient was diagnosed with a biopsy-proven multifocal glioblastoma. **D** Sagittal TSE, Short TI Inversion Recovery (STIR) T2-weighted, post-gadolinium TSE T1-weighted, and axial Gradient Echo (GE) T2-weighted images showed diffuse thickening of the cauda equina nerve roots. Mild enhancement was noted on T1-weighted images. The spinal canal diameter was normal, and no focal disk herniations were detected. The patient had non-Hodgkin lymphoma (NHL) and was diagnosed with neurolymphomatosis

Neoplastic disorders (*n* = 26) included metastasis from systemic cancer (*n* = 12, 46%), primary brain tumors (*n* = 10, 38%), and carcinomatous meningitis (*n* = 4, 15%). Imaging examples of patients with an alternative neoplastic diagnosis are presented in Fig. 2.

**Fig. 3:**
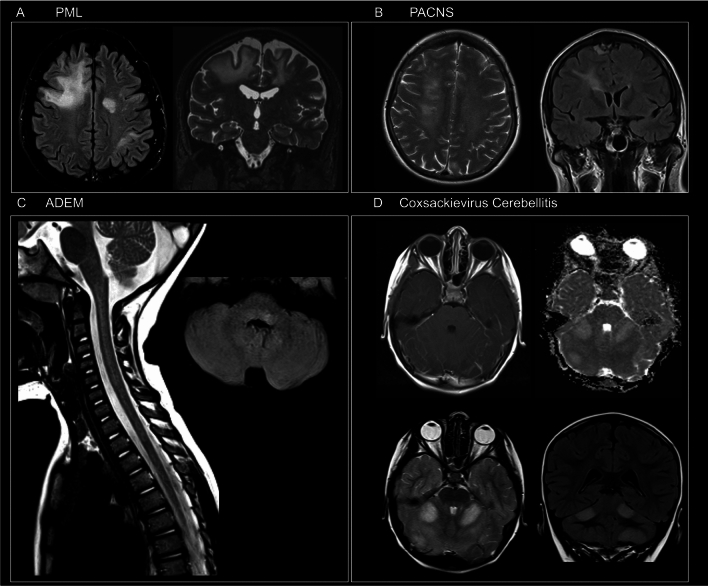
Imaging examples of patients who were initially thought to have PNS but later had an alternative autoimmune or infectious diagnosis. **A** Axial Fluid Attenuated Inversion Recovery (FLAIR) and coronal multiplanar reconstruction of volumetric Turbo Spin Echo (TSE) T2-weighted sequence showing multifocal, asymmetric, and bilateral hyperintensities involving deep and subcortical white matter of both cerebral hemispheres. The lesions were sparing the cortex and involved U-fibers. These findings were consistent with a classic form of progressive multifocal leukoencephalopathy (PML) which was later confirmed by JCV detection on cerebrospinal fluid. **B** Axial TSE T2-weighted and coronal FLAIR images demonstrating a hyperintense lesion extending from periventricular white matter to the corona radiata. On post-gadolinium Spin Echo (SE) T1-weighted images a focal, linear enhancement in the same area was present. Intense pachymeningeal enhancement and a dural nodule were also noted. All these findings were suggestive of central nervous system vasculitis. A diagnosis of primary angiitis of the central nervous system (PACNS) was made. **C** Sagittal TSE T2-weighted of cervical spine and axial FLAIR images showing a large T2-weighted hyperintensity lesion involving the cervical spinal cord for more than two metameres and a focal hyperintensity on the posterior side of the medulla on the left, consistent with a final diagnosis of acute disseminated encephalomyelitis (ADEM). **D** Multiple hyperintense T2-weighted focal lesions showing diffusion restriction [high signal on apparent diffusion coefficient (ADC) map] on both cerebellar hemispheres. A mild enhancement along cerebellar folia was seen. The patient had an infectious cerebellitis due to Coxsackie virus

Infectious diseases (*n* = 21) were infectious encephalitis or meningoencephalitis (*n* = 16, 76%), infectious myelitis (*n* = 2, 10%), septic encephalopathies (*n* = 2, 10%), and infectious (West Nile virus-associated) optic neuropathy (*n* = 1, 5%). Imaging examples of patients who were initially thought to have PNS but later had an alternative autoimmune or infectious diagnosis are presented in Fig. 3.

Patients with psychiatric diseases (*n* = 13) comprised functional neurological disorders (*n* = 5, 38%), primary psychosis (*n* = 4, 31%), or other psychiatric diseases (*n* = 4, 31%).

Toxic and metabolic disorders (*n* = 19) included cases of vitamin deficiency with neurological involvement (*n* = 6, 32%), alcohol-related CNS disorders (*n* = 4, 21%), neuroleptic side-effects (*n* = 2, 10%), and other less frequent toxicities (drug-related optic neuritis, radiation toxicity, and opioids overdose in 1 each) or metabolic disorders (multifactorial in 2, and hyponatremic encephalopathy and hepatic encephalopathy in 1 each).

### Alternative diagnoses with peripheral nervous system involvement

During the study period, the etiology was identified in 112/215 patients with PerNS involvement (52%), while in 103 (48%), it remained unknown. The most frequent etiologies were, in order of frequency: autoimmune 51 (45%), toxic-metabolic 40 (36%), and vascular 11 (10%), followed by other less frequent categories in 10 (9%) (Fig. 1).

Among disorders tested for PNS Abs with an autoimmune etiology (*n* = 51), Guillain-Barré syndrome (*n* = 20, 39%) was the most common diagnosis, with three patients presenting with an acute motor-sensory axonal neuropathy variant (AMSAN), while the others presented with the classic acute inflammatory demyelinating polyneuropathy form (AIDP). Three patients with GBS had a previous history of cancer. Chronic inflammatory demyelinating polyneuropathy (CIDP) was the second most common diagnosis in this group (*n* = 9, 18%), whereas the remainder of patients (*n* = 22, 43%) presented with peripheral nervous system involvement in rheumatic or systemic autoimmune disorders.

Toxic and metabolic disorders (*n* = 40) were the following most common etiologies, including mixed forms (*n* = 13, 32%), diabetic (*n* = 7, 17%), chemotherapy-induced (*n* = 7, 17%), alcohol-related neuropathy (*n* = 3, 7%), and drug-induced neuromyopathy (*n* = 3, 7%), followed by other less frequent disorders (*n* = 7, 17%).

Vascular etiology (*n* = 11) consisted exclusively of vasculitic involvement of the peripheral nervous system.

### Alternative diagnoses with extra-nervous system involvement

Diseases without nervous system involvement were identified in 49/661 patients (7%). Vertigo of peripheral origin was the most common etiology in this group (*n* = 10, 20%), and one of the reasons for Ab testing in few patients was the presence of a concomitant tumor (*n* = 4). Patients with established extra-NS neoplastic diseases who experienced general medical complications were the second most frequent category (*n* = 4, 8%). The third group was represented by infections not involving the nervous system (*n* = 3, 6%). Finally, one last group (*n* = 32) comprised patients presenting with heterogeneous clinical pictures (e.g., profound asthenia). These presentations could not be framed in a unifying clinical entity and therefore were considered together in this miscellaneous subgroup.

### Associations with cancer

Overall, 141 tumors were found among the 661 patients with alternative diagnoses considered in the present study (21%). In the CNS disorder group, an associated tumor was identified in 80 patients (20%), including in order of frequency breast (*n* = 24), hematological (*n* = 10), lung (*n* = 9), urinary tract (*n* = 9), and gastrointestinal (*n* = 7), followed by other less represented tumors (*n* = 21). In PerNS disorders, an associated tumor was identified in 45 patients (21%), including in order of frequency breast (*n* = 16), lung (*n* = 9), hematological (*n* = 7), gastrointestinal (*n* = 5), and others (*n* = 8). Finally, an associated neoplastic disease was found in 16 patients (32%) with extra-NS involvement, including in order of frequency breast cancer (*n* = 6), gastrointestinal (*n* = 3), lung (*n* = 2), urinary system (*n* = 2), and hematological (*n* = 2), followed by one other non-identified primary tumor. Overall, tumors were mostly diagnosed before the neurological syndrome, regardless of the neurological dysfunction location (CNS *n* = 55 [69%], PerNS *n* = 30 [67%], and Extra-NS *n* = 10 [62%]).

A clinical vignette illustrating a neurological syndrome in a patient with breast cancer that was ultimately attributed to an alternative diagnosis rather than PNS is presented in the Supplementary Information.

### Laboratory testing yield

Considering the definite diagnoses of PNS (detailed in [6]), yearly point estimates of diagnostic yield for Ab testing (definite PNS/total number of patients tested) showed a bimodal pattern, but no significant linear temporal variations were evident (*p* = 0.9 for trend). The yearly number of patients tested increased from 67 (2009) to 131 (2017), a 96% increase. However, diagnostic yield remained low (7% [CI 95% 2–16] vs. 8% [CI 95% 4–14], respectively), while cumulative diagnostic yield was 10% [CI 95% 8–12] during the study period. Among the 661 patients in which an alternative diagnosis was established, only 63 (10%) and 172 (26%) had an initial presentation suggestive of a high-risk or intermediate-risk PNS phenotype respectively, while 426 (64%) did not have a clinical presentation compatible with PNS according to the updated diagnostic criteria [2].

### Variable selection and score building

Results of the logistic regression model are shown in Table 1. Intermediate-risk syndromes or other neurological presentations (OR 7.97, CI95% 3.07–23.80 and OR 192.82 CI95% 66.11–739.59, respectively) and low-risk tumor/absence of tumor (OR 6.90, CI95% 2.75–18.92) were associated with higher odds of an alternative diagnosis than PNS, whereas increasing age (OR 0.95, CI95% 0.92–0.97) was inversely associated with an alternative diagnosis. A distribution of these variables according to the presence of an underlying etiological diagnosis is reported in Table S1. No separation was found among categorical variables in the score model, and therefore, no regularization method was used. However, we note that the size of some of the categorical variables was small as reported in Table S2. Calibration curves alongside Brier scores for the model in the discovery and validation cohorts are presented in Fig. S1. The *PNS DDx Score* was then derived and pooled into three categories identifying low (− 3 to 0), medium (1–3), and high (> 3) probability of an alternative diagnosis (Table 2). These cut-offs were chosen to maximize specificity.

**Table 1:** Logistic regression model evaluating OR (CI 95%) of an alternative diagnosis of PNS

		Outcome (alternative diagnosis)		
Predictors	Beta	OR	CI	*P*
Age	−0.05	0.95	0.92–0.97	< 0.001
Sex (female)	0.22	1.25	0.58–2.75	0.6
Neurological syndrome				
Intermediate-risk vs. high-risk^a^	2.07	7.97	3.07–23.80	< 0.001
Other vs. high-risk	5.26	192.82	66.11–739.59	< 0.001
Low-risk tumor/absence of tumor^b^	1.93	6.90	2.75–18.92	< 0.001
*R*^2^ = 0.548				

^a^Intermediate- and high-risk syndromes are defined in Table 3

^b^Low-risk tumors were those that did not belong to the typical oncological associations described in the updated PNS diagnostic criteria. Conversely, high-risk tumors included lung (both small-cell and non-small-cell), thymoma, breast and ovarian cancers, testicular cancer, and Hodgkin lymphoma

**Table 2:** *PNS DDx Score* items, range and point categories for low, medium, and high probability of an alternative diagnosis of PNS, respectively

Items	Sub-items	Points
Age (years)	< 55	0
	55–70	−2
	> 70	−3
Syndrome type	Other^a^	5
	Intermediate-risk^a^	2
	High-risk^a^	0
Tumor presence and risk	Low-risk^b^/No tumor	2
	High-risk	0
Range		−3 to 7
Probability of alternative diagnosis		Score categories
Low		−3 to 0
Medium		1 to 3
High		> 3

^a^“Other” syndromes include defined clinical phenotypes that are epidemiologically not associated with cancer (e.g., Parkinson disease and multiple sclerosis). Intermediate- and high-risk syndromes are defined in Table 3

^b^Low-risk tumors were those that did not belong to the typical oncological associations described in the updated PNS diagnostic criteria. Conversely, high-risk tumors included lung (both small-cell and non-small-cell), thymoma, breast and ovarian cancers, testicular cancer, and Hodgkin lymphoma

### *PNS Score* validation

Mean diagnostic accuracy in the derivation cohort was overall good (AUC 0.891, CI95% 0.883–0.971), while the validation cohort outperformed the training set (AUC 0.931, CI95% 0.929–0.997). However, this score was not validated in the possible/probable PNS diagnosis subgroup, as no definite outcome is available for this category. Additional performance metrics are reported in Table S3.

## Discussion

The spectrum of alternative diagnoses in patients suspected of having PNS is broad and encompasses both common and uncommon disorders, some of them treatable [2, 9]. Herein, CNS diseases in which either atypical clinical-laboratory features or an associated cancer led the clinician to perform neuronal Ab testing accounted for most cases. In addition, other cases were tested for neuronal Abs in the context of a diagnostic workup when a clear clinical diagnosis was not readily achievable. This approach is not advisable as PNS are rare disorders, in which the risk of false-positive Ab results is high [2, 11, 12]. Considering the low pretest probability of a true PNS diagnosis, this combination provides an overall high risk of misdiagnosis according to the Bayes theorem [16, 17]. This is the reason why the updated diagnostic criteria for PNS highlight the need of carefully considering neuronal Ab testing only in specific clinical scenarios, while avoiding its indiscriminate and unfocused use to prevent potential harm to the patients [2], as it was observed in a recent study focused on AE misdiagnosis [13].

The three most common etiologies in the setting of CNS diseases were degenerative (36%), autoimmune (17%), and vascular (14%): these categories, in fact, harbor disorders with clinical presentation and progression that may mimic PNS [13, 18–20]. However, the two most represented neurodegenerative phenotypes (dementias and MND) have very rarely a paraneoplastic origin. A recent study performed in the Netherlands which involved neuronal Ab testing in 920 patients with a diagnosis of neurodegenerative dementia found that only 7 (0.8%) harbored neural Abs (anti-IgLON5 in 3, anti-LGI1 in 2, anti-DPPX in 1, anti-NMDAR in 1), none of them belonging to the “high-risk” Ab group [19]. Importantly, all these 7 cases had atypical features for neurodegenerative dementia (e.g., subacute deterioration) [19]. Therefore, these findings and the observations from our study suggest that unselected neuronal Ab testing in patients with neurodegenerative dementia lacking atypical findings should not be routinely proposed. Regarding MND, only 1/878 patients (0.11%) had a definite PNS diagnosis while manifesting an MND-like phenotype [6]. In agreement with this finding, the previous studies showed that paraneoplastic MND-like phenotypes are rarely encountered, and these cases showed additional atypical features such as narcolepsy-cataplexy, hyperphagia and sexual dysfunction (in the case of anti-Ma2 PNS), other atypical non-motor neurological manifestations (in the case of anti-Hu PNS), or inflammatory CSF alterations (i.e., pleocytosis or presence of CSF-restricted oligoclonal bands) [21, 22]. Again, in the absence of such atypical features, neuronal Ab testing should not be performed in patients with MND phenotype.

Patients with isolated psychiatric disorders were also tested for neuronal Abs with negative results. This is in line with a previous prospective study that examined 105 patients with first episode psychosis (FEP), none of them harboring anti-NMDAR Abs [23], suggesting that in patients with FEP lacking neurologic symptoms and abnormalities on paraclinical studies (brain MRI and CSF examination), the probability of AE (including paraneoplastic AE) is probably very low. In this setting, we would like to underline the role of prolonged electroencephalogram in the diagnostic process, a non-invasive and inexpensive test which is abnormal in most patients with autoimmune encephalitis, while typically normal in most patients with primary psychiatric disorders [24].

Concerning PerNS diseases, the reason for neuronal Ab testing was either atypical clinical-laboratory features or associated tumors, but etiological diagnosis was achieved in only half of the cases, a much lower proportion than what seen in CNS disorder. This finding is consistent with prior reports demonstrating that a clear etiology is not identified in a substantial proportion of patients with polyneuropathy, even at tertiary referral centers [25]. Notably, re-evaluation of cases previously classified as idiopathic using advanced diagnostic modalities, including genetic testing and skin biopsy, has shown that approximately one-third remain without an identifiable cause. Furthermore, the inclusion of small-fiber neuropathy may have contributed to the relatively high proportion of cases with unknown etiology in this study. In PerNS diseases group, the three most common etiologies were autoimmune (45%), toxic-metabolic (36%), and vascular (10%). The most common PerNS disorders screened for the presence of neuronal Abs were Guillain-Barré syndrome (mostly of the typical AIDP variant) and CIDP, despite the fact that they are well-defined diseases not epidemiologically related to cancer, conditions in which current diagnostic criteria do not recommend neuronal Ab testing [2].

Interestingly, the proportion of patients with associated tumors was similar in both CNS and PerNS (20% and 21%, respectively), while it represented a higher share in extra-NS groups (32%), possibly implying that when the clinical picture is not definite, a positive neoplastic medical history determines a lower threshold for neuronal Ab testing. Nevertheless, a mere temporal association with cancer is not sufficient for a PNS diagnosis, which requires a causal association [2]. Indeed, patients with true PNS often harbor genetic alterations within the tumors that trigger the autoimmune reaction (often in the genes encoding for the antigenic target, such as CDR2L and CDR2 for the anti-Yo PNS [26], or associated proteins, such as KCTD16 for anti-GABA_B_R PNS [27]), while for other types of cancer (e.g., prostate or renal carcinoma), the association with a neurological syndrome should be carefully evaluated, while searching for other neoplasms [28]. More importantly, in true PNS, the neurological syndrome typically precedes—and often leads to—the diagnosis of an underlying tumor [2]. In contrast, in our cohort, patients ultimately found to have an alternative diagnosis more often had a prior history of cancer that prompted the search for neuronal Abs.

Diagnostic testing yield fluctuated in the study period considered, with an increasing number of patients tested over the years, possibly reflecting the increasing availability of commercial kits for neuronal Ab testing. The overall yield was estimated at 10%, similar to another recent study performed in the Netherlands (9.5%) [4], probably implying that there is an overuse of testing, also when not clinically indicated.

Considering the multitude of alternative diagnoses encountered in our population, it is difficult to extrapolate a diagnostic algorithm that can be reliably used in clinical practice. However, some major considerations and recommendations for neural Ab testing can be derived from the present study (Table 3).

**Table 3:** Recommendations for neuronal antibody testing in PNS

Clinical settings in which PNS neuronal Ab testing is indicated:
High-risk phenotypes
• Encephalomyelitis • Limbic encephalitis • Rapidly progressive cerebellar syndrome • Opsoclonus–myoclonus • Sensory neuronopathy • Gastrointestinal pseudo-obstruction • Lambert–Eaton myasthenic syndrome
Intermediate-risk phenotypes
• Autoimmune encephalitis • Brainstem encephalitis • Morvan syndrome • Stiff-person syndrome • Isolated myelopathy (only selected cases, e.g., those with longitudinally extensive, symmetric, tract specific abnormalities in spine MRI) • Polyradiculoneuropathies (only selected cases, e.g., those with concomitant involvement of CNS)
Clinical settings in which neural Ab testing is *not* indicated:
Defined phenotype epidemiologically not associated with cancer (e.g., multiple sclerosis, Parkinson’s disease, Alzheimer’s disease, Guillain-Barré syndrome, primary psychiatric disorders, etc.)
Red flags suggesting alternative diagnoses other than PNS
* Clinical:* onset in childhood, slow progression over several months/years, history of cancer at advanced disease stage or cancer usually not associated with PNS (e.g., prostate cancer), severe malnutrition, previous treatment with neurotoxic medications other than ICIs, normal neurologic examination, or examination in line with functional neurologic disorder
* Paraclinical:* lack of inflammatory alterations on CSF and/or MRI studies, presence of reduced glucose content or malignant cells on CSF examination, and normal EEG in patients with psychiatric disturbances

*Ab* antibody, *CNS* central nervous system, *CSF* cerebrospinal fluid, *EEG* electroencephalogram, *ICIs* immune checkpoint inhibitors, *MRI* magnetic resonance imaging, *PNS* paraneoplastic neurological syndromes

The *PNS DDx Score* should be interpreted as a support to the neurological examination and medical history in the clinical setting where PNS is suspected. We suggest that a *PNS DDx score* > 3 is associated with a high probability of an alternative diagnosis of PNS and should therefore refrain from second-level PNS diagnostics without a strong clinical suspicion. Nevertheless, a high *PNS DDx score* does not completely exclude a PNS diagnosis and is intended only as a tool in the diagnostic evaluation of neurological syndromes resembling PNS, to avoid unnecessary and potentially invasive diagnostic workup at an early stage.

Across diagnostic categories, PNS Ab testing was primarily ordered at the discretion of the treating physician in the presence of atypical clinical features or a known systemic malignancy, rather than in typical presentations suggestive of PNS. As a result, a major limitation of this study is that the spectrum of alternative diagnoses identified does not necessarily reflect the true differential diagnosis or misdiagnosis spectrum of PNS as encountered in specialized referral centers. Instead, these findings likely capture real-world testing practices, in which neuronal Ab testing is frequently applied outside recommended indications, leading to substantial overtesting. Such practice patterns have important clinical implications, including potential delays in establishing the correct diagnosis—particularly when alternative conditions are amenable to treatment—as well as unnecessary downstream investigations, such as repeated cancer screening, increased health care utilization, and patient anxiety.

Additional limitations should be acknowledged. First, paraneoplastic antibody assays evolved during the study period (2009–2017), which may have influenced Ab detection and subsequent diagnostic attribution. Second, although cancer screening was performed according to contemporary standards, the extent and duration of longitudinal tumor surveillance may have varied across patients, potentially affecting exclusion of an underlying malignancy in selected cases. Third, due to the retrospective design of the study, standardization of clinical, neuroradiological, and laboratory follow-up was not possible and was based on the treating clinician’s judgment. We also acknowledge that our study did not systematically collect data on all red flags and they are only partially included in the score. However, the scope of our score was to provide a simple clinical tool to guide antibody testing in the setting of suspected PNS, while many of the red flags imply a more advanced diagnostic workup. Finally, our results need to be replicated in an external independent validation before translation into clinical practice. However, we note that our study cohort reflects laboratory referral from three independent clinical centers (Udine, Pordenone, and Gorizia), thus supporting the clinical applicability of the score.

Despite these limitations, this was the first study examining in detail the spectrum of alternative diagnoses encountered during PNS workup, which provides useful information in guiding neuronal Ab testing in more selected clinical settings. Educational activities focused on PNS diagnosis are probably needed for neurologists and oncologists caring for these patients.

## Conclusions

During PNS diagnostic evaluation, many alternative diagnoses (“better explanations”) are identified, encompassing both central and peripheral nervous system disorders. These disorders are particularly common in patients < 55 years and those without high-risk syndromes. The *PNS DDx Score*, based on age, syndrome, and tumor type, can be used to identify patients at risk of PNS misdiagnosis. Further studies will be needed to refine Ab testing indications and avoid improper testing in the era of broader availability of Ab diagnostic kits.

## Supplementary Information

Below is the link to the electronic supplementary material.

Supplementary file1 (DOCX 4277 KB)
