# Supplementary material for: Alternative diagnoses of suspected paraneoplastic neurological syndromes: a population-based study
Source: J Neurol. 2026 Mar 11;273(3):200. doi: 10.1007/s00415-026-13737-w (PMC12979337; doi:10.1007/s00415-026-13737-w)
Supplement: Supplementary file 1 — Supplementary file1 (DOCX 4277 KB) [file 415_2026_13737_MOESM1_ESM.docx]

**Supplementary Information**

**Manuscript Title:** Alternative diagnoses of suspected paraneoplastic neurological syndromes: a population-based study

- Table S1
- Table S2
- Table S3
- Figure S1
- Clinical vignette

**Table S1:** Distribution of the main score variables in patients with known and unknown underlying etiological diagnosis according to nervous system involvement (central vs peripheral).

| **Variable** | **CNS (n=397)** | | **P value *** | **PerNS (n=215)** | | **P value *** |
| --- | --- | --- | --- | --- | --- | --- |
| **Etiology** | **Known (n=269)** | **Unknown (n=128)** |  | **Known**  **(n=112)** | **Unknown (n=103)** |  |
| Age (y), mean (SD) | 61.9 (18.5) | 57.7 (18.7) | **0.04** | 67.4 (12.5) | 65.5 (15.4) | 0.3 |
| Neurological syndrome, n (%)  *High risk*  *Intermediate risk*  *Other* | 44 (16)  58 (22)  167 (62) | 12 (9)  6 (5)  110 (86) | **<0.001** | 2 (2)  69 (62)  41 (37) | 3 (3)  38 (37)  62 (60) | **0.001** |
| Tumor presence, n (%) | 53 (20) | 27 (21) | 0.7 | 30 (27) | 15 (15) | **0.03** |

**Legend:** CNS=Central Nervous System; PerNS=Peripheral Nervous System. * Student’s t-test for continuous variable, Chi-square test for categorical variables.

**Table S2:** Distribution of categorical variables in the score model according to the study outcome (derivation cohort only).

| **Categorical Variables, n (%)** | **Outcome** | |
| --- | --- | --- |
|  | **Alternative diagnosis n=460** | **Definite diagnosis**  **N=65** |
| **Sex (Female)** | 233 (51%) | 35 (53%) |
| **Neurological syndromes** |  |  |
| *Classic* | 50 (11%) | 55 (85%) |
| *Non-classic* | 40 (9%) | 6 (9%) |
| *Other* | 370 (80%) | 4 (6%) |
| **Tumor** |  |  |
| *Low risk/Absence* | 401 (87%) | 36 (55%) |
| *High risk* | 59 (13%) | 29 (45%) |

**Table S3:** Performance metrics for PNS DDx score.

|  | **Discovery cohort** | **Validation cohort** |
| --- | --- | --- |
| AUC (CI 95%) | 0.891 (0.883; 0.971) | 0.931(0.929; 0.997) |
| Sensitivity (CI 95%) | 86.3% (83.7; 98.3) | 95.0% (92.0-99.0) |
| Specificity (CI 95%) | 90.8% (78.5; 96.9) | 95.8% (87.5-100) |
| PPV (CI 95%) | 98.5% (96.8; 99.5) | 99.5% (98.4-100) |
| NPV (CI 95%) | 48.4% (43.9; 86.6) | 69.7% (60.0-91.7) |
| Accuracy (CI 95%) | 86.9% (84.6; 96.2) | 95.1% (92.4-98.7) |

**Legend:** AUC=Area Under the Curve; PPV=Positive Predictive Value; NPV=Negative Predictive Value. CI 95%= Confidence Interval 95%.

**Figure S1**: Calibration curves for the score model in the discovery (A) and validation (B) cohorts.


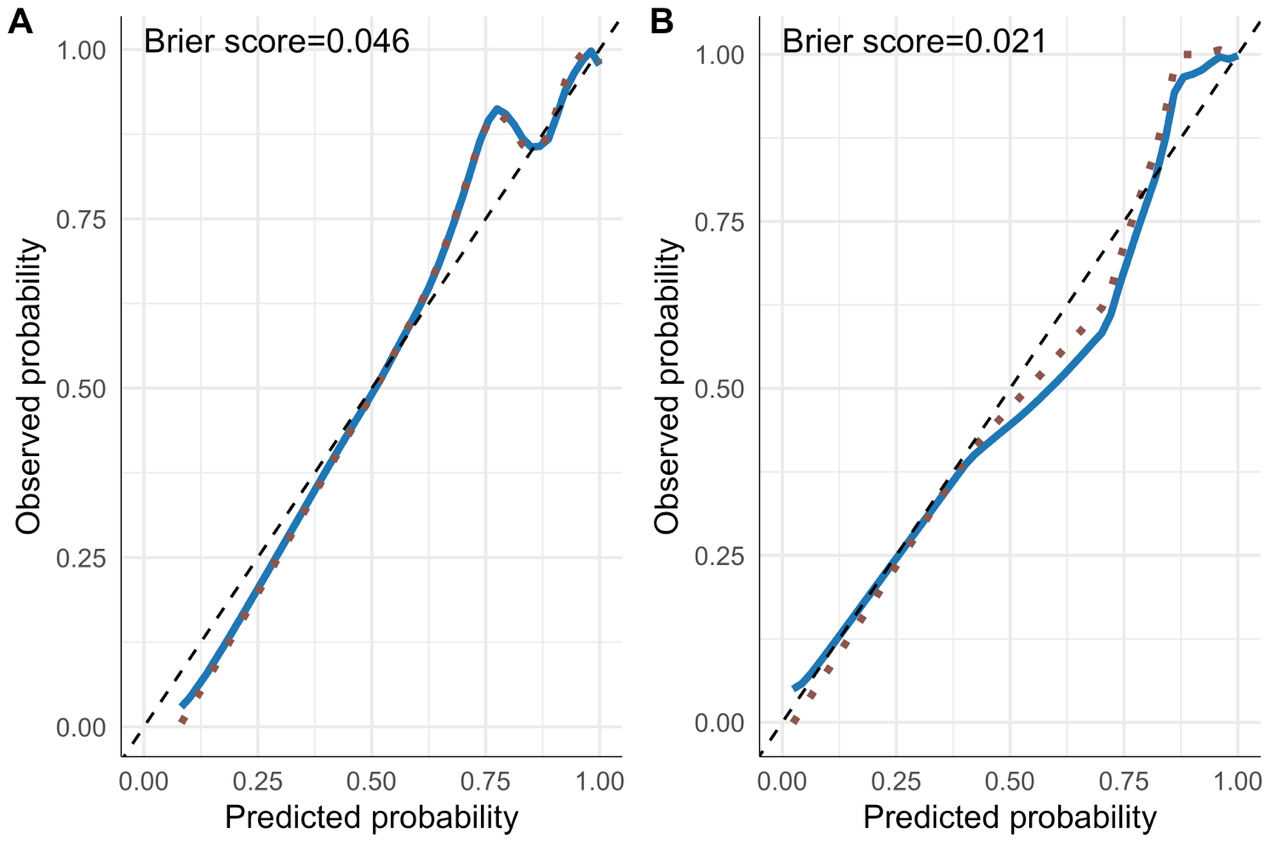


**Legend:** Black dashed line=ideal line; Red-dotted=Naive line; Blue line=Bias corrected line.

**Clinical vignette**

A 75-year-old woman with a previous history of breast cancer presented with a slowly progressive neurologic syndrome characterized by autonomic failure and parkinsonism. The disease onset was marked by symptomatic orthostatic hypotension and urinary dysfunction, followed by the development of bradykinesia with rigidity and minimal response to levodopa therapy. Brain MRI demonstrated infratentorial atrophy. Testing for PNS Abs was negative. Based on the overall clinical presentation and diagnostic findings, a diagnosis of multiple system atrophy, parkinsonian type (MSA-P), was made. The patient did not fulfill criteria for a PNS diagnosis.
